# Validation of the Ischaemia Severity Scale (ISS) Based on Non-Invasive Vascular Assessments (SEWSS) for Predicting Outcomes of Diabetic Foot Attack

**DOI:** 10.3390/jcm11237195

**Published:** 2022-12-03

**Authors:** Fermín Rafael Martinez-De Jesús, Emmanuel Hernandez-Luevano, Neftalí Rodriguez-Ramírez, Rafael Cendejas-Alatorre, José Antonio Muñoa Prado, Favio Carrera Maigua, Elízabeth Zambrano-Loaiza

**Affiliations:** 1 The Diabetic Foot Latinamerican Society Research Group 1, Diabetic Foot Salvage and Prevention Center Saint Elian, Veracruz 91900, Mexico; 2 The Diabetic Foot Latinamerican Society Research Group 2, Diabetic Foot Salvage and Prevention Center Saint Elian, Veracruz 91900, Mexico

**Keywords:** wound healing, amputations, ischaemia, diabetic foot, ankle/brachial index, toe/brachial index

## Abstract

Assessment of ischaemia severity includes a variety of measures, such as pedal pulse palpation, the ankle/brachial index (ABI), and the toe/brachial index (TBI), but there is a lack of consensus regarding which ischaemia scale is the most effective for determining outcome prognosis. The purpose of this study is to validate the application of the ischaemia severity scale (ISS) in the effective prediction of wound healing, amputations, and mortality for diabetic foot wounds (DFW). This prospective study included 235 consecutive patients graded according to the Saint Elian Wound Score System (SEWSS). The ISS is part of this system, with patients being scored as non-ischaemic (0) or having mild (1), moderate (2), or severe (3) ischaemia. Age, diabetes duration in years, and ulcer size were found to be associated with a longer mean ischaemia of increasing severity. A trend of reduction in the pulse palpation rates (70.4%, 50%, 8.5% to 0%; *p* < 0.01), ABI (1.1 ± 0.1, 0.86 ± 0.3, 0.68 ± 0.2, 0.47 ± 0.2, *p* < 0.01), TBI average values (0.90 ± 0.35, 0.62 ± 0.52, 0.50 ± 0.33, 0.10 ± 0.42, *p* < 0.01), wound healing success (88.7%, 57.7%, 40.7%, 12.9%; *p* < 0.01), and delay in weeks (Kaplan–Meier: log-rank 44.2, *p* < 0.01) was observed with increasing values of the ISS (0, 1, 2, and 3). The odds ratio for adverse outcomes increased for each additional level of ischaemia severity. Thus, we demonstrate that the ISS is useful in effectively predicting adverse outcomes for DFW.

## 1. Introduction

Diabetic foot syndrome is defined as an acute or chronic attack characterised by one or more foot wounds that can differ in aetiology, complexity, and severity grade factors, including extent, depth, anatomic zones and aspect locations, infection, ischaemia, oedema, and neuropathy, and it is associated with increased amputations and death risk in persons with diabetes [1]. Foot wounds are among the most complex and heterogeneous complications in diabetic patients. A foot wound is defined as a breakdown in the protective function of the skin below the ankle in a diabetic patient, irrespective of its aetiology or duration. In our previous study [2], we demonstrated that the outcomes for ischaemia are a relevant part of comprehensive severity wound scoring for variables that positively or negatively affect wound healing progress. Ischaemia is included among the prognosis factors scored and graded homogeneously, from mild to severe, as part of this classification system. Ischaemia has the worst prognosis of the ten severity factors assessed for wound healing progress, amputations, and death in diabetic foot patients. Patients with ischaemia of varying severity have increased odds ratios for major amputations and wound healing failure compared with non-ischaemic patients [2]. The assessment of ischaemia in a clinical setting includes questioning and clinical examination in combination with a variety of measures, such as pedal pulse palpation, the ankle/brachial index (ABI), the toe/brachial index (TBI), and waveform analysis, but there is no consensus regarding which assessment method is the most effective for diagnosis [3,4]. The ABI is a very useful clinical index for assessing arterial blood supply to the foot, but there are limitations when used in consideration of people with diabetes [4]. Medial calcification in diabetes, known as Mönckeberg’s sclerosis, causes the incompressibility of the foot arteries, which might affect the accuracy of the ankle/brachial index [5,6]. Autonomic neuropathy and chronic renal insufficiency are highly associated with Mönckeberg’s sclerosis [7]. Interval ABI and TBI results are used to monitor the efficacy of revascularisation procedures in the lower extremities and to predict wound healing and future cardiovascular-related morbidity and mortality [8]. There is limited research on the reliability of these non-invasive vascular tests in patients with varying stages of diabetic foot wounds. We hypothesised that a comprehensive diagnostic approach with the addition of several tests, including a register of the Doppler waveform analysis, would improve the accuracy of prognoses, so we used this approach for the assessment of the ischaemia severity level as part of the system for classifying diabetic foot wound severity [2]. In this study, we validate the application of the severity grade scores for ischaemia that are included in wound severity classification for predicting the wound healing rate, major amputations, deaths, and treatment abandonment in patients with diabetic foot wounds.

## 2. Materials and Methods

Consecutive patients with type 2 diabetes and diabetic foot wounds who arrived for care at our centre were initially included in the study.

### 2.1. Primary Objective and Measurements

The primary endpoint was the rates of wound healing, major amputations, deaths, and patient treatment abandonment for each level of the ischaemia severity scale (ISS) in patients with diabetic foot wounds. All patients provided written informed consent, and the Human Subjects Ethics Committee reviewed and approved the study.

The inclusion criteria were patients with type 2 diabetes who were over 18 years of age and had wounds at or distal to the malleoli with different degrees of infection, oedema, ischaemia, neuropathy, anatomic factors, and tissue affection. The extent and depth of tissue death was included as part of each wound assessment. Wound size was measured as the maximum length by the maximum width. Wounds secondary to gangrene debridement or after the surgical removal of infected tissue were included. Exclusion criteria included a diagnosis of severe cardiovascular or renal failure or severe neurological problems that would make the patient a poor candidate for the study (e.g., being bedridden). Patients with no family assistance were also excluded from the study.

### 2.2. Demographic, Clinical, and Wound Characteristics

Baseline demographic measurements were performed at the first visit. The diagnosis of diabetes was made prior to enrolment. The severity and type of diabetic foot attack was immediately diagnosed at presentation. Oedema, neuropathy, vascular, and infection assessments and wound characteristics were included and assessed according to the Saint Elian Wound Score System for diabetic foot attack. This system uses the following ten wound variables categories, which are measured at patient presentation: (1) primary location, (2) topographic aspects, (3) number of affected zones, (4) ischaemia, (5) infection, (6) oedema, (7) neuropathy, (8) depth, (9) area, and (10) wound healing phase. Each factor was subcategorised using an ascending severity score from mild (1 point) to severe (3 points). The score sum was a maximum of 30 points. A score sum of 10 points or fewer was graded as I (mild; successful wound healing likely). A moderate score of 11 to 20 points was graded as II (partial foot threatening; outcome related to “state-of-the-art” therapies used and associated with a good patient biological response), and 21 to 30 points was graded as III (limb- and life-threatening; outcome unrelated to “state-of-the-art” therapies due to poor biological patient response).

### 2.3. Non-Invasive Vascular Assessment to Categorise Severity Grades of Ischaemia (ISS)

Ischaemia levels of mild (1 point), moderate (2), and severe (3) were categorised following non-invasive vascular assessment. We chose the test with a reputed higher accuracy when there were any discrepancies between the results of one or more methods used to scale ischaemia severity. The evidence-based predictive values escalated from pedal pulse palpations to ABI, TBI, and waveform pulse analysis. Doppler waveform analysis with graphic report was performed only in patients with ischaemia. Patients were diagnosed, clinically or according to the ABI, TBI, and waveform analysis results, for subcategorisation as ischaemic (scaled 1 to 3) or non-ischaemic (scaled as zero) patients.

#### 2.3.1. Pulse Palpation

Two different members of staff determined ischaemia severity according to the scale using the dorsalis pedis and tibialis posterior arteries of the foot: palpation of a bound strong arterial pulse (0, non-ischaemic), palpable but slightly diminished (1, mild), thready and scarcely palpable (2, moderate), and non-palpable pulses (3, severe).

#### 2.3.2. Ankle/Brachial and Toe/Brachial Index

All participants were required to lay supine for a minimum of ten minutes prior to any assessment, and systolic pressure was measured. The tibialis posterior and dorsalis pedis artery pressures were assessed and used for the ABI calculation (Hand-held Doppler–Huntleigh, Getinge AB; 8 MHz Doppler probe). A regular pulse was found, and the sphygmomanometer was pumped up slowly to a maximum of 200 mmHg to occlude digital blood flow. The systolic pressure was obtained by deflating the cuff. Toe pressure was measured using a 2.5 cm wide × 9 cm long digital cuff on the proximal aspect of the hallux to calculate the TBI. A PPG unit Hadeco Smartdop 30EX was used when no toe artery was found using a Doppler. The toe/brachial index and ABI were determined by dividing the higher systolic pressure of the toe or ankle with the maximum blood pressure of the arms. The ABI and TBI were separately calculated for each leg, and the measurement of the wounded limb of the two values was taken as the result for the study patient. Ischaemia was defined as an ABI < 0.9 and TBI < 0.75. The following range categories of the ABI for the ISS were used: 0.9 to 1.2 (0, normal), 0.7 to 0.89 (1, mild), 0.5 to 0.69 (2, moderate), and <0.50 (3, severe). The following TBI levels for the ISS were used: >0.75 (0, normal), 0.60 to 0.74 (1, mild), 0.30 to 0.59 (2, moderate), and <0.30 (3, severe).

#### 2.3.3. Index Agreement

Two members of staff independently conducted each non-invasive test for the assessment of the ISS. The kappa agreement index was calculated using 2 × 2 tables to detect differences between the two observers. A kappa value (K ¼ Po-Pe/1-Pe; Po ¼ observed agreement, Pe ¼ expected agreement by random) between 0.61 and 1.0 indicates substantial to excellent agreement.

### 2.4. Standard of Care Treatment and Therapeutic Intervention

All patients were treated using an outpatient ambulatory model, which included appropriate surgical debridement, aggressive parenteral/intramuscular broad-spectrum antimicrobial administration, appropriate off-loading, and strict glycaemic control. Angioplasty and bypass were performed in patients with ischaemia who accepted the procedure. All patients were initially followed on a daily basis and, depending on the condition of the wound, were seen every third day or once weekly. Cardiovascular disease, nephropathy, retinopathy, and neurological problems were assessed. The follow-up period was part of the normal treatment duration according to the healing success or failure plus a secondary follow-up within a minimum of 6 months for delayed wound healing. The patient was deemed to have completed the study once total ulcer healing was achieved. Early and direct deaths were those that occurred as a consequence of the diabetic foot wounds during therapy or within 30 days after. Delayed mortality occurred after this early period as a consequence of conditions other than an acute diabetic foot problem. Consecutive score measurements were recorded at different dates. Daily wound images and wound measurements were recorded in a database for systematic data collection.

Significance was considered at *p* < 0.05. Values of chi-square with Yate’s correction or the Fisher’s exact test with 2 × 2 tables or Mantel–Haenszel χ^2^ for linear trends, and variance ratios for natural and treatment analysis of variance, were calculated. Kaplan–Meier probability, log-rank test, and Cox hazard ratio analyses were performed for wound healing time and measured against the study’s ischaemia severity scores. The study population included every patient who was consecutively assessed by their random presentation. Because the entire population was included, it was not necessary to calculate the sample size.

## 3. Results

### 3.1. Patients

A total of 235 patients with type 2 diabetes and diabetic foot wounds who consecutively presented for care at our centre were initially assessed and included in the study. Each patient was invited to participate in the study at presentation and informed that the assessment was part of their diagnosis and treatment and was being performed for research purposes. Outcomes were assessed during a mean follow-up period of 52 weeks, which was extended to 156 weeks for assessing delayed mortality. The treatment period duration was 9.3 ± 9.2 weeks (range 1 to 30 weeks).

### 3.2. Demographic, Clinical, and Wound Characteristics

According to the ischaemia severity scale, there were 159 non-ischaemic patients (67.6%), 24 (10.2%) patients with mild ischaemia, 25 (10.6%) patients with moderate ischaemia, and 27 (11.4%) patients with severe ischaemia. There were no differences due to gender, smoking, HbA1c, or wound history in weeks. Age, diabetes duration in years, ulcer size, and severity wound score average exhibited an increasing trend for increasing grades of ischaemia severity (Table 1). Ischaemia occurred at a very low rate in patients at SEWSS grade I with a mild severity score (8.3%) and successful wound healing, and no patient with this grade was observed when the ISS was moderate and severe. However, at Saint Elian severity wound grade II (partially foot-threatening), there were no differences in patient ratings for any ISS. A significant ascending trend of ISS patient ratings of severity wound grade III (limb- and life-threatening) was observed (Table 1). The ten categories of Saint Elian classification.

The severity factor scores of the Saint Elian classification exhibited similar proportions (*p* > 0.05) to the Ischaemia Severity Score (from zero to three), aside from in the number of affected zones and the area size grades (*p* < 0.05). The percentages of patients with small wound sizes diminished as the severity of ischaemia increased (83.6%, 70.8%, 64.5%, and 44.4% from 0 to 3, respectively), and this was also the case for medium (10.1%, 12.5%, 16%, and 37%, respectively) and large (6.3%, 16.7%, 20%, and 18.5%, respectively) wound sizes (*p* < 0.01). There was a descending trend in ischaemia rates for one (76.4%, 10.8%, 6.8%, and 6.1%, respectively), two (56.3%, 9.4%, 18.8%, and 15.6%, respectively), and three affected foot zones (43.5%, 8.7%, 13%, and 34.8%, respectively). Non-ischaemic patients exhibited a decreasing trend in ischaemia rates from one to two and three affected foot zones (71.1%, 22.6%, and 6.3%, respectively). Conversely, patients with increasing ischaemia severity exhibited an increasing number of affected zones.

#### Non-Invasive Vascular Tests to Categorise Severity Grades for Ischaemia

A pulse palpation test was performed, and the ABI calculated for 100% of the study patients (30% resulted with Mönckeberg’s sclerosis). The TBI was calculated in 88.1% of the population because no pulse was found or because there was a previous amputation or a wound involving the first ray. Doppler waveform analysis with graphic reporting was performed in 81 patients (34.4%). All patients were submitted to angioplasty or bypass (15.5%), including Doppler waveform analysis, as part of the surgical protocol to assess the arterial flow starting at the femoral common level. The pulse palpation rates of the foot arteries revealed a decreasing trend when progressing from non-ischaemic patients to a mild, moderate, and severe grade of ischaemia. There were no pulse palpations on the dorsalis pedis and tibialis posterior arteries when ischaemia was categorised as severe. The systolic ankle pressures were 124 ± 44.4, 125 ± 58.7, and 95 ± 61.8 and toe pressures were 91.5 ± 62.3, 81.3 ± 77.4, and 26.5 ± 48.2 for mild, moderate, and severe grades, respectively (*p* < 0.01). A reducing trend in the average ankle and toe/brachial index values with increasing ischaemia severity grades was confirmed. In total, 81 of the 235 (34.4%) patients who were previously diagnosed as patients with ischaemia could be differentiated based on their ISS levels when including pulse palpation and the ABI and TBI values. Pulse waveform analysis confirmed the absence of ischaemia with a normal triphasic wave in five of these patients (3.1%) and different grades for ischaemia in 76 of these patients (96.9%). No normal triphasic waveforms were recorded for any ISS score for ischaemia severity. A biphasic waveform was prevalent for mild ischaemia. A monophasic waveform was prevalent at a moderate score (76%). No pulse wave was recorded for severe ischaemia in 81.4% of the patients. The test results to categorise ischaemia revealed significant differences in non-ischaemic patients compared to patients with ascending grades of ischaemia severity (Table 2).

### 3.3. Kappa Agreement Index

The observers showed high agreement, 0.91 and 0.94, for the categorisation of normal pulse palpation, wherein non-ischaemic patients scored 0, and the absence of pulse palpation (scored as 3 points) in severe ischaemic patients, respectively. The agreement scores descended to 0.7 and 0.68 for mild and moderate ischaemia, respectively, based on scoring by pulse. The ABI, TBI, and waveform pulse recordings showed a kappa agreement index above 0.78 for all levels of the ischaemia severity scale.

#### Outcomes for Primary Endpoints

**Figure 1:**
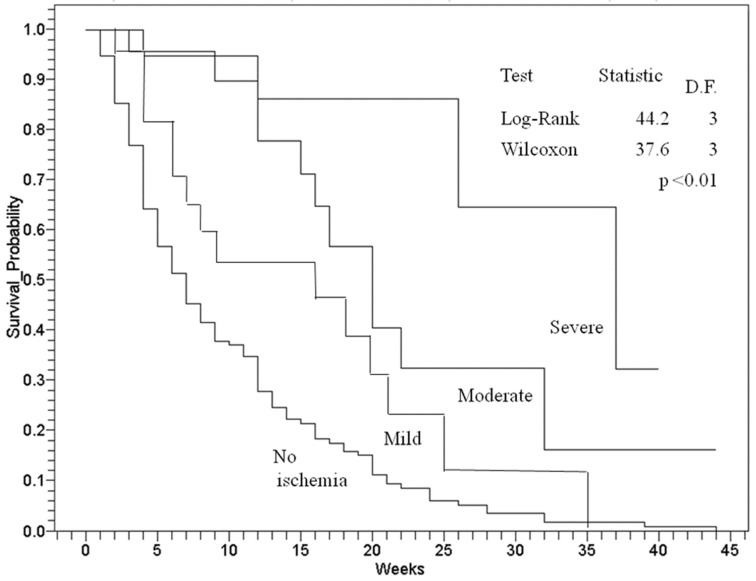
Kaplan–Meier survival probability analysis for wound healing failure by weeks according to the severity of ischaemia.

Wound healing success and failure were validated according to the subcategories for ischaemia. The Kaplan–Meier survival probability analysis for wound healing failure by weeks demonstrated that the delay in the wound healing time differed according to the graded ischaemia severity (Figure 1).

The Cox proportional hazard scores demonstrate that the survival probability for wound healing success was significantly higher for mild ischaemia (hazard ratio: 9.5; 95% CI 2.1–41.2, *p* < 0.01) and non-ischaemic patients (hazard ratio: 13.8; 95% CI 3.4 to 56.1, *p* < 0.01) than for severe ischaemia (likelihood ratio of 52; *p* < 0.05). The hazard ratio was not significantly different when comparing moderate and severe ischaemia (*p* < 0.05). Wound healing rates decreased significantly according to the severity of ischaemia, and the levels of amputations and patient abandonment of treatment increased (Table 3).

The probabilities of healing failure, major amputations, delayed death, and treatment abandonment were determined using odds ratio calculations of the ischaemia severity grades against non-ischaemic patients (Table 3) with diabetic foot wounds (likelihood ratios of 79.7, 60.5, 25, and 15.1, respectively). The odds ratio for delayed mortality as a consequence of kidney or cardiac failure or stroke increased significantly as the ischaemia severity grade increased, but the Cox hazard ratios were not significantly different. Early mortality as a direct consequence of diabetic foot was not significant and found to be independent of the ischaemia severity scale.

## 4. Discussion

The results of this study confirm that there are differences between scales according to ischaemia severity grades. The range of score subcategories from non-ischaemic patients, to mild, moderate, and severe ischaemia (0–3), was predetermined by a review of published values for pedal pulses, the ABI, TBI, and waveform analysis. A variance or failure of concordance of the scores of each test used to categorise the ISS was expected as an alternative to the null hypothesis. However, the characterisation of ischaemia severity levels was validated by the hard data obtained from the non-invasive tests in our study. The variability in the published reports in terms of the reliability of these tests is controversial, particularly with regard to their predictive value when used as a single test. Most of these studies were designed to evaluate sensitivity and specificity to detect the percentage of arterial stenosis using angiography as the “gold standard” [8]. Reliability in measuring perfusion distal to the ankle is required to adequately assess and treat patients with diabetic foot, where the concern is to determine ischaemia severity as a predictor of wound healing and amputation outcomes. Angiography is an invasive test that is not currently used for diagnosis, but it is fundamental in planning the surgical approach when necessary. Angiograms are not safe, and possible complications include allergic reactions to the contrast dye, damage to blood vessels, blood clots, bleeding, and kidney damage, particularly for patients with diabetes and whose kidney functions are already impaired. Knowledge of the length, level, and number of stenoses provided by angiography in the diabetic foot is needed to plan and perform the corrective surgical procedure after diagnosis, and this level of arterial obstruction was achieved using vascular non-invasive tests. Therefore, we performed predictive analysis using discrete choice models based on logistic regression (odds and likelihood ratios) and the survival probability of Kaplan–Meier analysis for non-healing patients. The reliability may be improved by adding the values of different non-invasive tests to increase the accuracy of clinical judgements. The palpation of pedal pulses is a subjective measurement, and the palpation of pulses becomes increasingly difficult as ischaemia becomes more severe. There is also large inter-rater variability among inexperienced clinicians when determining the palpation of pedal pulses [3,9,10,11], but pedal pulse palpation is the only way to assess the arterial perfusion of the feet in many primary care settings. Our study provides data to support the application of a pulse palpation assessment of the ischaemia severity scale in selecting patients for referral to a vascular or diabetic foot unit or deciding to continue their care at the same level. The ABI is a reliable measure of peripheral arterial disease in patients without diabetes, with excellent sensitivity and specificity [12]. However, the ABI has limited applicability in patients with long-standing diabetes because of the likelihood of falsely elevated readings [3,7]. Neither the Society for Vascular Technology [13] nor a consensus paper [14] explain how the limits of the ABI range were derived. The ranges for the ABI appear to be derived from original data in Yao [13], Cornwall [14], and Sumner [15]. Numerous methods of calculating the ABI have been described [16,17,18,19,20] based on variances in the numerator in the ABI equation: (a) the current method uses the high ankle pressure (HAP) of the two ankle systolic arterial pressures as the numerator in the ABI equation [18]; (b) a second method uses the low ankle pressure (LAP) of the two ankle systolic arterial pressures as the numerator [17,18]; (c) a third calculation uses the average of the two ankle systolic pressures as the numerator in the ABI equation [18]; (d) a few studies have used the tibialis posterior artery systolic pressure to calculate the ABI [20]. We used the HAP to calculate the ABI based on the dorsalis pedal or tibialis posterior arteries. A comparison of the four assessment methods is underway to determine which ABI modalities are superior in predicting outcomes. The limitations to using the ABI are that the ABI is age- and blood pressure-dependent, it encompasses the arterial flow of the anterior and tibialis posterior arteries, and it does not identify any occlusion or calcification of vessels distal to this site [21]. Calculating the toe brachial index (TBI) solves these problems [21], but it is not feasible to register the digital pulses in some patients if there is a previous amputation or a wound at the first toe (11.9% in the present study). Using a Doppler probe, toe pulses were not detected in 28 patients (15%), and the use of the PPG unit Hadeco Smartdop was a useful alternative [22]. Toe pressure and the TBI may be used as an adjunct to a standard peripheral arterial assessment performed by general practitioners, podiatrists, vascular surgeons, and nurses to obtain quantitative baseline measures or to confirm the diagnosis and severity of ischaemia in diabetic foot patients. The pulse palpation, the ankle brachial pressure index, and the toe brachial pressure index were useful for assessing the severity of ischaemia. Our results validate the ISS with non-invasive assessments, which must be available for health care professionals in primary settings. Our study shows that toe pressure, as a non-costly test, is easy to determine for use in grading ischaemia severity, increasing its potential when combined with pulse palpation, plethysmography, and pulse wave registration. If the primary care setting lacks these sets of tests, then pulse palpation provides a reasonable alternative to the ISS scale. In one classification [23], the scale for ischaemia was validated according to TcPo2, the determination of which is expensive and frequently unavailable at diabetic foot centres. We were unable to measure transcutaneous oxygen pressures (TcPO2), as the diagnostic kit for this was not available for our patients. We were advised of its variability and would suggest it has value as part of a scoring system rather than in isolation. As part of the WiFi classification, TcpO2 fails to be superior over toe pressure measurements for haemodynamic monitoring during endovascular revascularisation [24]. Future research must clarify the impacts of these systems in health care for the prevention of amputations due to diabetic foot attack secondary to ischaemia.

The results of the present study clearly differentiate non-ischaemic from ischaemic patients and their severity grades. Non-ischaemic patients were found to be younger and have shorter diabetes duration and the smallest wound sizes in cm^2^. Patients with ischaemia revealed ascending severity scores for age, diabetes duration, and wound size, which negatively affected the prognoses for wound healing, amputations, and deaths. The Kaplan–Meier analysis and odds ratio results confirm differences in wound healing failure according to ischaemia severity grades, which may be explained as a consequence of the “poor” biological response of these patients, caused by the ageing process and body damage secondary to a longer duration of diabetes. The study population included every patient who was consecutively assessed by their random presentation. The entire population, and not a sample, was submitted to Kaplan–Meier analysis to avoid violations of the test assumptions. Ischaemia severity correlated with the Saint Elian severity grades with a high impact on outcomes, which were positive for grade I and negative for grade III. The odds ratio for direct early mortality secondary to a diabetic foot wound was not significant, but there is an increase in the OR for delayed mortality according to the ISS in the follow-up. Our report is in accordance with the current evidence for the use of the ABI in the identification of patients at high risk of future cardiovascular and cerebrovascular mortality [24,25]. A systematic review of 11 published studies on 44,590 subjects was performed by Heald et al. [26], who reported that an ABI < 0.9 is associated with an increase in all causes of mortality, cardiovascular and cerebrovascular mortality, coronary heart disease, and fatal and non-fatal stroke. In our study, we confirmed that the odds ratio increases for delayed mortality (cardiovascular, renal, or stroke) accordingly with the ischaemia severity grades in persons with diabetes and diabetic foot wounds.

## 5. Conclusions

Our results may assist health care professionals in wound care within a diabetic foot clinic or at primary care facilities through using the ISS to select the most appropriate treatments based on the probability of healing in patients with wounds along with monitoring. The Saint Elian score [1,2,27,28,29] for severity wound grades provides a platform for prevention, diagnosis, and prognosis. Aggravating factors, such as oedema, infection, neuropathy (Charcot) [30], and ischaemia, must be independently assessed and treated in therapeutic decisions. These treatments may involve revascularisation, amputation, or conservative management. The ischaemia severity scale provides the clinician with a better understanding of healing potential and whether there is an opportunity for the vascular team to improve the flow to the extremities using revascularisation techniques, such as angioplasty or bypass surgery. The ISS is useful for predicting adverse outcomes of DFW.

## Figures and Tables

**Table 1:** Baseline demographic, clinical, and wound characteristics.

	Ischaemia Severity Scale				
	0	1	2	3	
	No	Mild	Moderate	Severe	
Characteristic	*n* = 159	*n* = 24	*n* = 25	*n* = 27	*p* Value
Age (y) +	61.3 ± 11.1	71.9 ± 9.5	69 ± 10.5	71.3 ± 7.3	<0.01
Gender **					
Male	68 (42.8)	14 (58.3)	13 (52)	14 (51.9)	0.21
Female	91 (57.2)	10 (41.7)	12 (48)	13 (48.1)	
Diabetes duration in years +	17.7 ± 8.9	22.6 ± 10.9	19.8 ± 11.5	24.1 ± 12.7	0.03
HbA1c +	8.6 ± 2.3	7.4 ± 2.3	8.0 ± 1.9	7.8 ± 2.1	0.19
Smoking **	52 (33)	6 (25)	6 (24)	7 (26)	0.29
Wound history (weeks) +	5.8 ± 7.5	8.6 ± 9.8	6.6 ± 8.8	7.5 ± 5.8	0.41
Wound size cm^2^	11.3 ± 36.4	13.9 ± 22.3	19.8 ± 36.5	19.1 ± 18.9	<0.01
Saint Elian score means ± SD +	14.6 ± 3.7	15.6 ± 4.0	17.8 ± 3.6	19.3 ± 3.2	<0.01
Saint Elian wound severity grades **					
I (good prognosis for wound healing)	23 (14.5)	2 (8.3)	0	0	0.03
II (partially foot-threatening)	124 (78)	18 (75)	18 (72)	18 (66.7)	0.16
III (limb- and life-threatening)	12 (7.5)	4 (16.7)	7 (28)	9 (33.3)	<0.01

Values are the mean ± SD or actual (percent); ** Mantel–Haenszel chi square for trends or + Kruskal–Wallis.

**Table 2:** Non-invasive vascular assessment to categorise ischaemia according to grade of severity.

	Ischaemia Severity Scale				
	0	1	2	3	
	No	Mild	Moderate	Severe	
Non-Invasive Vascular Tests	*n* = 159	*n* = 24	*n* = 25	*n* = 27	*p* Value
Pulses palpation of foot arteries					
Dorsalis pedis	112 (70.4)	12 (50)	2 (8.5)	0	<0.01 *
Tibialis posterior	103 (64.7)	10 (41.6)	1 (4)	0	<0.01 *
Ankle/brachial index	1.1 ± 0.1	0.86 ± 0.3	0.68 ± 0.2	0.47 ± 0.2	0.01 +
Toe/brachial index	0.90 ± 0.35	0.62 ± 0.52	0.50 ± 0.33	0.10 ± 0.42	<0.01 +

Values are the mean ± SD or actual (percent); * Mantel–Haenszel chi-square for trends or + Kruskal–Wallis.

**Table 3:** Outcome rates and odds ratio for ischaemia severity scale in diabetic foot patients.

Ischaemia Severity Scale				
Outcomes	0 No *n* = 159	1 Mild *n* = 24	2 Moderate *n* = 25	3 Severe *n* = 27
Wound healing *	134 (88.7)	15 (57.7) [2.7, 1.0–7.5]	11 (40.7) [9.5, 3.8–23.5]	4 (12.9) [74.4, 16.5–335]
Major amputation *	7 (4.4)	3 (12.5) [3.4, 0.8–14.4]	4 (16) [3.9, 1.0–14.8]	19 (70.3) [51.9, 17–59.2]
Abandonment of treatment *	28 (17.6)	7 (29.2) ** [2.0, 0.7–5.3] ***	9 (36) [2.7, 1.1–6.5]	13 (48.1) [4.6, 1.9–10.9]
Early deaths **	3 (1.9)	0	2 (8) [4.2, 0.6–26.9]	1 (3.7) [1.9, 0.1–19.7]

Wound healing: percentages are for success and odds ratio for failure. Values are actual (percent) and odds ratio [OR, 95% C.I.]. * Significant values for Mantel–Haenszel chi-squared for trend, ** non-significant values. All odds ratio values are significant for logistic regression, except *** non-significant value.

## Data Availability

Data supporting the reported results can be found within the archives of the clinical charts in our Centre.
